# Increased shoulder pain across an exercise session and subsequent shoulder exercise: a prospective cohort study

**DOI:** 10.1186/s12891-022-05674-2

**Published:** 2022-07-29

**Authors:** Jeanette Trøstrup, Susanne Wulff Svendsen, Annett Dalbøge, Lone Ramer Mikkelsen, Mette Terp Høybye, Lene Bastrup Jørgensen, Thomas Martin Klebe, Poul Frost

**Affiliations:** 1 Elective Surgery Centre, Silkeborg Regional Hospital, 8600 Silkeborg, Denmark; 2 Department of Clinical Medicine, Aarhus University, 8200 Aarhus N, Denmark; 3 Danish Ramazzini Centre, Department of Occupational Medicine, Gødstrup Hospital – University Research Clinic, 7400 Herning, Denmark; 4 Danish Ramazzini Centre, Department of Occupational Medicine, Aarhus University Hospital, 8200 Aarhus N, Denmark; 5 Knowledge Centre for Neurorehabilitation of Western Denmark, Regional Hospital Central Jutland, 8450 Hammel, Denmark; 6 Centre for Social Medicine, Bispebjerg and Frederiksberg Hospital, 2000 Frederiksberg, Denmark

**Keywords:** Adherence, Fear-avoidance beliefs, Rehabilitation

## Abstract

**Background:**

Shoulder complaints are common and the recommended first-line treatment is exercise therapy. However, it remains unknown if increased shoulder pain after an exercise session is a barrier for subsequent exercise dose, particularly in people with high fear-avoidance beliefs. Such knowledge could indicate ways to optimise shoulder rehabilitation. The aim was to examine whether increased shoulder pain across an exercise session was associated with a lower subsequent exercise dose, and if high fear-avoidance beliefs exaggerated this association.

**Methods:**

We conducted a prospective cohort study using data from a randomised controlled trial in Central Denmark Region 2017–2019. Participants were employees (*n* = 79) with shoulder complaints and high occupational shoulder exposures. The intervention was a home-based or partly supervised exercise programme lasting 2–3 months. Linear mixed models were used to examine the associations between change in shoulder pain and exercise dose (number of repetitions, progression level (1–3), resistance level (1–3), and time until next exercise session [days]).

**Results:**

At baseline, the participants had a median pain intensity at rest of 2 on a numerical rating scale (0–10). For a 1-cm increase in pain on a visual analogue scale (0–10 cm) during an exercise session, the subsequent number of repetitions, progression level and resistance level were − 1.3 (95% confidence interval [CI] − 3.4 to 0.9), 0.0 (95% CI − 0.1 to 0.0) and − 0.0 (95% CI − 0.1 to 0.0), respectively. Likewise, the time until next exercise session was − 0.6 (95% CI − 2.4 to 1.3) days for a 1-cm increase. There were no interactions with fear-avoidance beliefs.

**Conclusion:**

Increased pain across an exercise session was not associated with subsequent exercise dose, regardless of fear-avoidance beliefs, among employees with shoulder complaints and high occupational shoulder exposures.

**Trial registration:**

The trial was registered at Clinicaltrials.gov 19/05/2017 (ID: NCT03159910).

**Supplementary Information:**

The online version contains supplementary material available at 10.1186/s12891-022-05674-2.

## Background

Shoulder complaints are common in the adult population, with a 1-month prevalence of 19–31%, where the variation primarily depended on case definitions [1]. Exercise therapy is recommended as first-line treatment [2–5], and supervised and home-based exercise seem equally effective [5–8]. During exercise therapy, aggravation of shoulder pain should be kept to a minimum [3] and the exercise dose in the subsequent session should be reduced if pain aggravation does not subside shortly after exercising [3, 9, 10]. Two views exist regarding the accepted degree of shoulder pain aggravation during exercise therapy [3, 8]. One group argues that pain aggravation may indicate overload of stressed tissues because of too difficult exercises or too high exercise load and decrease exercise motivation [3]; another group argues that pain aggravation may guide exercise progression and increase exercise motivation [3]. Persons who exercise to reduce shoulder complaints report lower motivation if the exercise feels harmful, but higher motivation if the exercise feels beneficial [11, 12].

According to the fear-avoidance model, negative appraisals of pain may cause people to avoid physical activities, including exercise therapy, in order to reduce pain, but through a vicious circle, this may instead lead to increased pain and disability [13–15]. Two studies of patients with shoulder complaints treated by exercises [16, 17] showed an advantage of low fear-avoidance beliefs with respect to pain and function, whereas a third study [18] showed no prognostic role of fear-avoidance beliefs with respect to disability. The influence of fear-avoidance beliefs on exercise dose has not been investigated, but higher levels of fear-avoidance beliefs were associated with a higher probability of quitting an exercise intervention in people with non-specific chronic neck and shoulder pain [16].

We are not aware of studies that have examined whether increased shoulder pain after an exercise session is a barrier for subsequent exercise dose or adherence to exercise programmes, particularly in people with high fear-avoidance beliefs. Such knowledge could indicate ways to optimise shoulder rehabilitation.

The primary aim of this study was to examine whether increased shoulder pain across an exercise session was associated with a lower exercise dose in the subsequent session, and if this association (if any) was exaggerated by high levels of fear-avoidance beliefs. The secondary aim was to examine whether increased shoulder pain across exercise sessions together with high levels of fear-avoidance beliefs influenced adherence to the exercise programme.

## Methods

### Design and setting

We conducted a prospective cohort study. The study was a secondary analysis of data from a cluster-randomised controlled trial, which compared two interventions (Shoulder-Café and Shoulder-Guidance) to reduce shoulder complaints among employees with high occupational shoulder exposures (ID: NCT03159910 at Clinicaltrials.gov on 19/05/2017) [19]. Both groups were analysed as a cohort to answer the present research questions. The Danish Data Protection Agency (case number: 1–16-02–498-16) and the Committee on Health Research Ethics in Central Denmark Region approved the study (case number: 1–10-72–271-16). All participants provided written informed consent. We reported the study using the Strengthening the Reporting of Observational Studies in Epidemiology (STROBE) statement. According to the randomisation, which was performed at company level, participants were enrolled in an intervention between August 2017 and August 2019. In-person meetings took place at six municipal health centres in Central Denmark Region. All participants completed a baseline questionnaire before the randomisation result was revealed to them. An intervention period of 12 weeks was intended, but due to work schedules of the physiotherapists and holidays, the periods varied.

### Study population

Detailed in- and exclusion criteria for the randomised trial have been described previously [19]. In brief, the participants were 18–65 years of age, employed in occupations with high mechanical shoulder exposures (i.e., service, manufacturing and construction), experienced shoulder pain, were without previous shoulder surgery and had an Oxford Shoulder Score (OSS) ≤ 40. The OSS is a 12-item patient-reported tool to measure shoulder pain and function ranging from 0 (worst) to 48 (best) [20–22]. To be included in this study, data was required on change in pain and subsequent exercise dose.

### Interventions

All participants were advised to follow a home-based shoulder exercise programme described in a pamphlet [19]. The programme was based on effect of published exercise programmes [9, 23–26], and included elements known to motivate exercise adherence (few exercises [3, 27], progression with individual adjustments [3] and elastic bands making exercise possible everywhere [12, 28]. The 15-min programme was recommended to be followed three to four times per week throughout the intervention. In addition to one posture correction exercise, the programme comprised three resistance exercises performed bilaterally with an elastic band: two exercises for the scapula stabilising muscles and one for the rotator cuff muscles. Each resistance exercise had three progression levels and three resistance levels (elastic band) (low = 1, medium = 2 and high = 3) (Additional file). Participants were recommended to start with the lowest progression and resistance levels, and to perform as many repetitions as possible until they were able to perform more than 3 sets of 15 repetitions. At this point, they were advised to progress the exercises. Progression included using an elastic band with higher resistance and to go on to the next progression level when the highest resistance level was reached. Participants were informed that pain aggravation (without further specification) during exercise could be expected, but if the aggravation did not cease within 1 h after the exercise session, they were recommended to decrease one progression or resistance level, or to decrease the number of repetitions in the next exercise session [19].

Additionally, the participants in the Shoulder-Café group were offered three supervised exercise sessions in accordance with the exercise pamphlet, and a clinical shoulder examination performed by a physiotherapist [19]. Based on the clinical shoulder examination, subacromial impingement syndrome was considered to be present if anterolateral shoulder pain was accompanied by a positive result of at least three of the following five clinical tests: Hawkins’ test, Neer’s clinical test, painful arc test, Jobe’s test and pain on resisted external rotation [29, 30].

### Outcomes

Exercise dose was quantified in terms of: number of repetitions (total number of repetitions per exercise session), progression level (mean progression level per exercise session [mean across exercise sets per session]), resistance level (mean elastic band resistance per exercise session [mean across exercise sets per session]) and time until next exercise session (days between two exercise sessions). Adherence to the exercise programme was classified as high or low (≥ 2 vs < 2 weekly exercise sessions). Information on exercise dose and adherence was obtained via an exercise diary (Additional file). To describe exercise progression (as another aspect of adherence), we aggregated the number of exercises per participant during the first (1 to 7 weeks) and last (8 to 15 weeks) part of the intervention.

### Predictors

Change in shoulder pain was calculated as shoulder pain at rest shortly after an exercise session minus shoulder pain at rest shortly before the exercise session using a visual analogue scale (range 0 cm [no pain] to 10 cm [worst imaginable pain]) [31]. A positive change indicated an increase in pain. For descriptive purposes, we defined decreased shoulder pain as a change of <  − 1 cm, unchanged shoulder pain as a change of − 1 to 1 cm, and increased shoulder pain as a change of > 1 cm. The choice of these definitions was based on the observed distribution of changes in shoulder pain across exercise sessions. Information about pain was obtained from the exercise diary (Additional file).

Fear-avoidance beliefs were assessed using a shoulder version of the Fear-Avoidance Beliefs Questionnaire—Physical Activity (FABQ-PA) [32–34], which ranges from 0 [no fear-avoidance] to 24 [high fear-avoidance]. The baseline FABQ-PA was used as a dichotomised score (low ≤ 14, high > 14) [35, 36]. Information about FABQ-PA was obtained from the baseline questionnaire.

### Covariates

Potential confounders comprised age, sex, body mass index (BMI), smoking status, dominant-sided pain, baseline pain at rest (measured with a numerical rating scale [NRS] ranging from 0 [no pain] to 10 [worst imaginable pain]), intervention group, days since start of intervention and session number. Age, sex, BMI and smoking status were included based on the literature [37, 38]. Apart from intervention group, days since start of intervention and session number, information on the covariates was collected through the baseline questionnaire.

### Statistical analyses

Continuous variables were presented as means with standard deviations (SD) or medians with interquartile ranges (IQR) depending on their distributions, and categorical variables as numbers and percentages. In descriptive analyses, missing values for number of repetitions, progression level and resistance level were replaced by values from the most recent exercise session with non-missing values, except for the first session, where missing values were replaced by values from the subsequent session. Remaining missing values were not replaced.

The associations between change in shoulder pain (continuous) and subsequent exercise dose (continuous) were analysed using linear mixed models allowing for clustering of data according to company and repeated measurements. Participants with a minimum of one exercise session including data for change in shoulder pain and one subsequent exercise session were kept in the models. The analyses were performed using crude and adjusted models including age (continuous), sex, BMI (continuous), smoking status (never, ex, current), dominant-sided pain (yes, no), baseline pain at rest (continuous), intervention group, days since start of intervention (continuous), session number (continuous) and an interaction term between change in shoulder pain (continuous) and baseline FABQ-PA (high, low). Associations were presented as mean differences with 95% confidence intervals (CI) based on bootstrapping (with 100 replications) to allow for non-normality of the outcome.

The influence of increased shoulder pain (continuous) and baseline FABQ-PA (high, low) on adherence (high, low) was analysed using logistic regression with robust standard errors allowing for intragroup correlation at company level. The analyses were performed using crude and adjusted models including the same covariates as listed above. Adherence was presented as odds ratio (OR) with 95% CI. We applied likelihood ratio test of no interaction between change in shoulder pain and high FABQ-PA. All analyses were performed using Stata 16 (StataCorp LP, College Station, TX, USA).

## Results

**Fig. 1:**
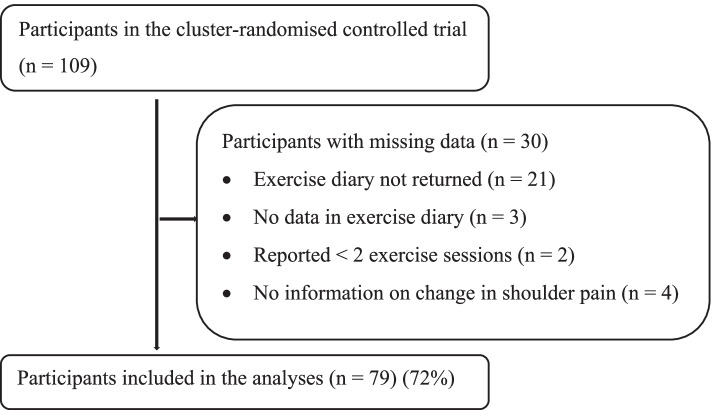
Flowchart

Out of the 109 participants in the randomised trial, 79 could be included (Fig. 1). Table 1 presents baseline characteristics of the participants included in the present study and those excluded due to missing data on change in pain and subsequent training dose. For the participants with a high FABQ-PA, the mean (SD) was 18.5 (2.5) for those included and 18.5 (3.0) for those with missing data. For participants with a low FABQ-PA the mean was 9.2 (4.0) for those included and 9.0 (3.8) for those with missing data. Pain duration ranged from around 1 to around 360 months. In the Shoulder-Café group, subacromial impingement syndrome was diagnosed in 38% (17/45) of the included participants and in 42% (5/12) of the participants with missing data.

**Table 1:** Participant baseline characteristics according to analysis status (*n* = 109)

Characteristics	Participants included in the analyses (*n* = 79)	Participants with missing data (*n* = 30)
Age (years), mean (SD)	48.0 (10.3)	45.5 (9.9)
Men, n (%)	51 (65)	23 (77)
BMI, mean (SD)	26.9 (4.9)	27.5 (8.4)
Occupation		
Service	21 (27)	7 (23)
Manufacturing	8 (10)	5 (17)
Construction	50 (63)	18 (60)
Smoking status, n (%)		
Never	37 (47)	13 (44)
Ex	28 (35)	7 (23)
Current	14 (18)	10 (33)
Dominant-sided pain, n (%)	54 (68)	24 (80)
High FABQ-PA^b^, n (%)	19 (24)	10 (34)^a^
NRS at rest, median (IQR)	2 (1 to 3)	3 (1 to 4)
High NRS at rest (NRS)^c^, n (%)	32 (41)	16 (53)
NRS during activity, median (IQR)	4 (2 to 6)	5 (2 to 7)
Pain duration (months), median (IQR)	39 (24 to 78)	69 (21 to 108)

*Abbreviations*: *BMI* Body mass index, *FABQ-PA* Fear-Avoidance Beliefs Questionnaire—Physical Activity (0 [no fear-avoidance] to 24 [high fear-avoidance]), *IQR* Interquartile range, *NRS* Numerical Rating Scale (0 [no pain] – 10 [worst imaginable pain]), *SD* Standard deviation

^a^FABQ-PA was missing for 1 participant in this group

^b^FABQ-PA was dichotomised as low (≤ 14) and high (> 14)

^c^Baseline pain at rest was dichotomised at the median (2, IQR 1–3) for all participants in the cluster-randomised controlled trial (*n* = 109)

**Fig. 2:**
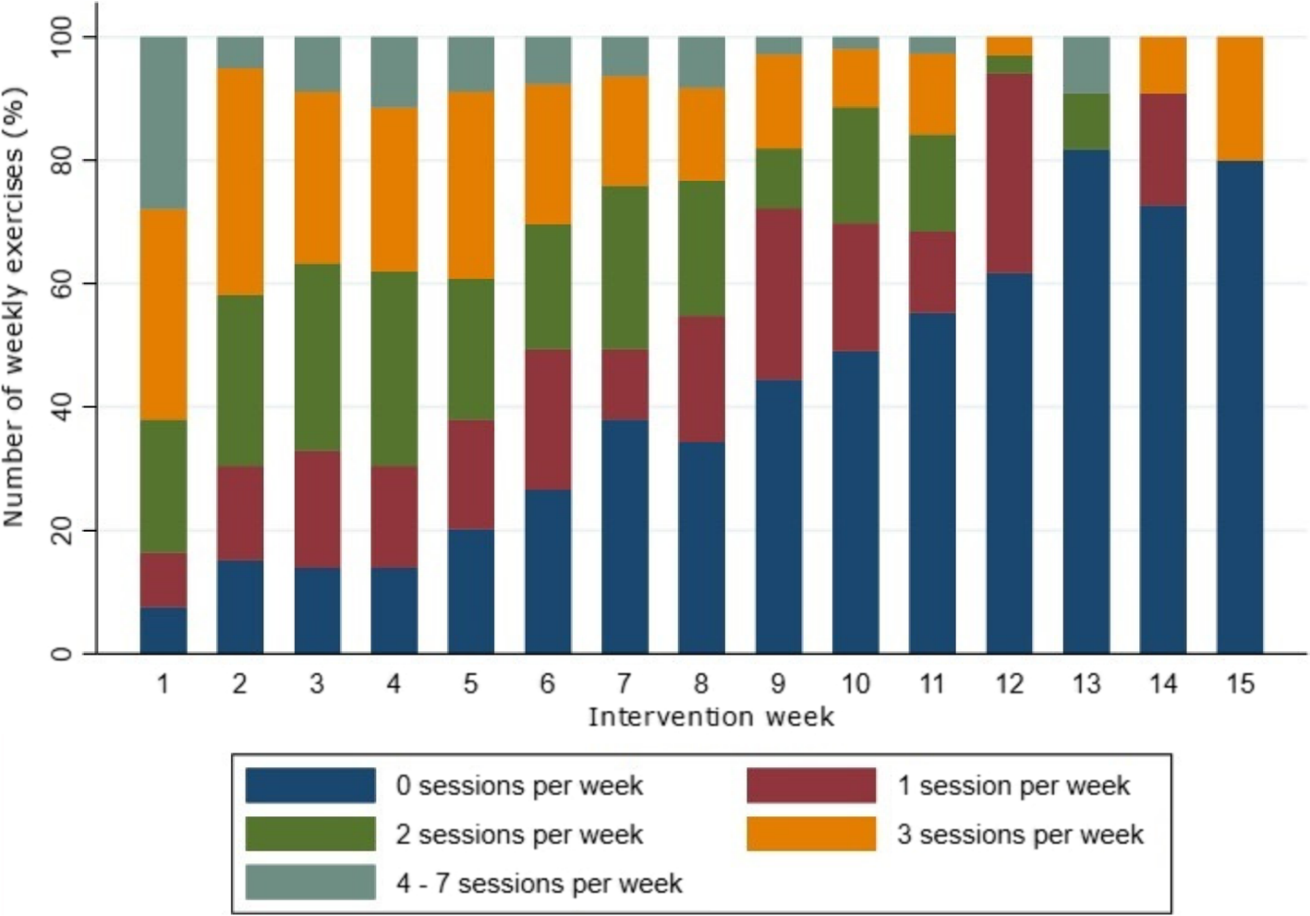
Distribution of weekly exercise sessions according to intervention week (*n* = 79). The participants performed a total of 1401 exercise sessions during 850 intervention weeks. The number of participants under intervention decreased gradually from 79 in week 7 to 5 in week 15

The intervention period was between 7 and 15 weeks in which the home-based exercises should be followed. In the Shoulder-Café group, 96% (43/45) additionally participated in two and 67% (30/45) in three supervised exercise sessions. The total number of intervention weeks among all participants was 850, during which a total of 1401 exercise sessions was performed. This corresponds to a mean of 17.7 (range: 2 to 50) exercise sessions per participant and a mean of 1.6 (range: 0 to 7) weekly exercise sessions. Figure 2 shows the distribution of weekly exercise sessions according to intervention week. The percentage of participants performing weekly exercises decreased gradually during the intervention period while the percentage performing zero weekly exercise sessions increased likewise.

Table 2 presents characteristics of the 1401 exercise sessions according to the first (1 to 7 weeks) and last (8 to 15 weeks) part of the intervention. There were no indications of exercise progression between the two parts.

**Table 2:** Characteristics of 1401 exercise sessions performed during intervention (*n* = 79)

Number of exercise sessions in relation to the two intervention periods^a^	Number of repetitions, mean (SD)	Progression level (range 1 to 3), mean (SD)	Resistance level (range 1 to 3), mean (SD)	Time until next exercise session (days), median (IQR)
Intervention weeks 1 to 7 (*n* = 1102)	124.4 (31.7)	2.1 (0.5)	2.0 (0.6)	2.7 (2.3 to 3.4)
Intervention weeks 8 to 15 (*n* = 299)^b^	118.7 (35.0)	2.2 (0.4)	2.0 (0.7)	2.6 (2.3 to 3.4)

*Abbreviations*: *IQR* Interquartile range, *SD* Standard deviation

^a^Between 3 and 23 missing values for number of repetitions, progression level and resistance level were replaced by values from the prior or subsequent exercise sessions

^b^The intervention period ended after 8–15 weeks

**Fig. 3:**
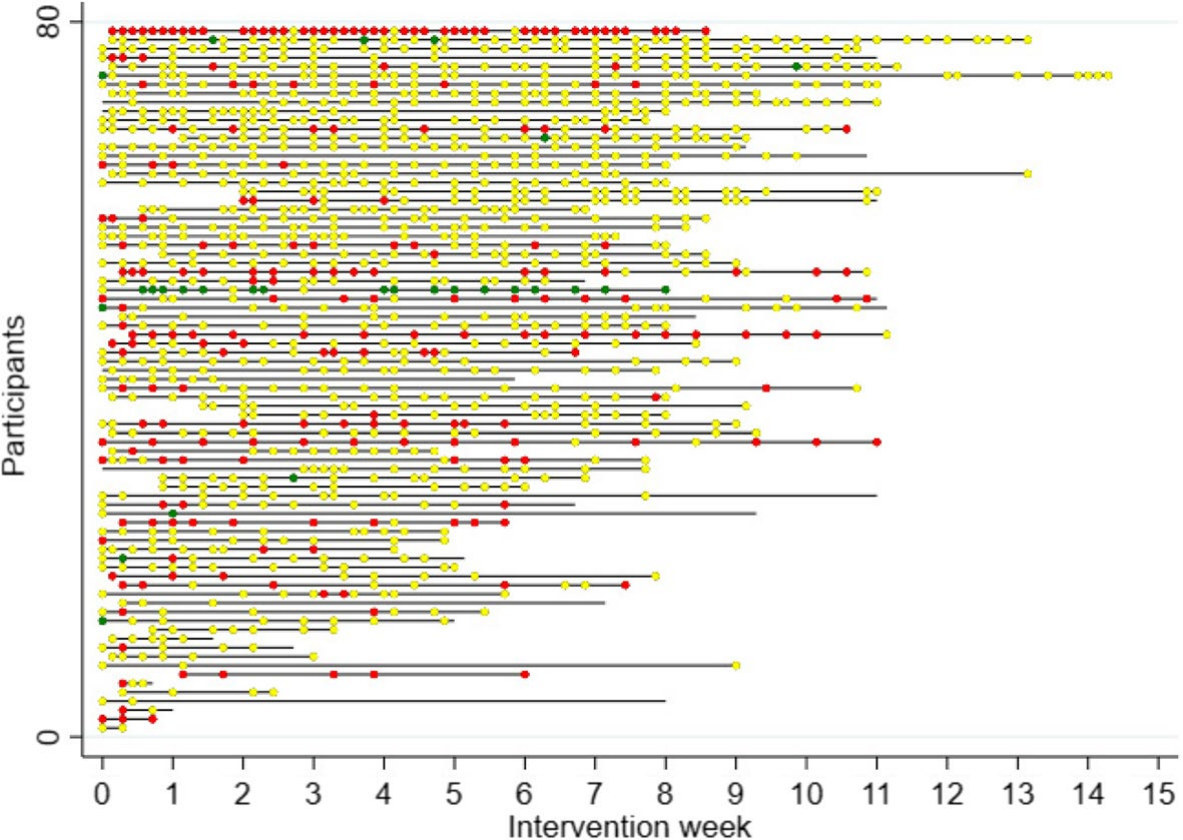
Distribution of pain change across exercise sessions (*n* = 1260) performed by 79 participants according to intervention week. The 79 participants were sorted according to the number of sessions they had performed. They performed 1260 exercise sessions in total. Green dots (*n* = 28) represent reduced pain after an exercise session (a change of <  − 1 cm), yellow dots (*n* = 1003) represent unchanged pain (a change of − 1 to 1 cm) and red dots (*n* = 229) represent increased pain (a change of > 1 cm)

Change in shoulder pain was missing for 141 sessions, leaving 1260 exercise sessions for further analyses. The mean shoulder pain shortly before and shortly after an exercise session was 1.6 (SD 1.5) and 1.9 (SD 1.8), respectively. Figure 3 illustrates the distribution of the 1260 exercise sessions, showing that unchanged pain was most common (80%). Reduced pain was found after 2% of the exercise sessions and increased pain after 18% of the sessions.

Out of the 1260 exercise sessions, 59 sessions were not followed by further sessions, leaving 1201 sessions for the analyses of associations between change in shoulder pain and subsequent exercise dose. As seen in Table 3 (adjusted results), increased shoulder pain across an exercise session did not influence subsequent exercise dose, and no interaction between change in shoulder pain and high FABQ-PA was found.

**Table 3:** Linear mixed models for shoulder pain and subsequent exercise dose, taking fear-avoidance beliefs into account. The analyses were based on 1201 exercise sessions (with information on subsequent exercise dose) performed by 79 participants

Predictors	Number of repetitions			Progression level			Resistance level			Time until next session (days)		
	Crude	Adjusted^a^		Crude	Adjusted^a^		Crude	Adjusted^a^		Crude	Adjusted^a^	
	MD	MD	95% CI	MD	MD	95% CI	MD	MD	95% CI	MD	MD	95% CI
Change in shoulder pain (for a 1-cm increase in VAS)	− 0.7	− 1.3	− 3.4 to 0.9	− 0.1^b^	-0.0	− 0.1 to 0.0	− 0.1^b^	− 0.0	− 0.1 to 0.0	− 0.4	− 0.6	− 2.4 to 1.3
High FABQ-PA	− 2.3	− 5.7	− 28.5 to 17.1	0.1	0.1	− 0.2 to 0.4	0.1	0.1	− 0.3 to 0.5	0.3	0.0	− 0.8 to 0.8
Interaction between change in shoulder pain and high FABQ-PA		0.2	− 6.8 to 7.1		− 0.0	− 0.2 to 0.1		− 0.1	− 0.2 to 0.1		0.5	− 1.7 to 2.7

*Abbreviations*: *CI* Confidence interval, *FABQ-PA* Fear-Avoidance Beliefs Questionnaire – Physical Activity (dichotomized; low ≤ 14; high > 14).), *MD* Mean difference, *VAS* Visual Analogue Scale (0 [no pain] – 10 [worst imaginable pain])

^a^Adjusted for age, sex, body mass index, smoking status, dominant-sided pain, baseline pain at rest, intervention group, days since start of intervention and session number with an interaction term between change in shoulder pain and fear-avoidance beliefs

bSignificant association (*p* < 0.05)

Thirty-one participants had high adherence. Table 4 shows that change in shoulder pain and high FABQ-PA did not influence adherence to the exercise programme (adjusted odds ratio [95% CI]: 0.6 [0.2 to 1.4] and 1.2 [0.4 to 4.3]). The likelihood ratio test had a *p*-value of 0.12 for no interaction between change in shoulder pain and high FABQ-PA.

**Table 4:** Logistic regression analysis of high adherence to the exercise programme. The analyses were based on 1401 exercise sessions performed by 79 participants. Estimates are odds ratios (OR) with 95% confidence intervals (CI)

Predictor	High adherence^a^			
	Crude		Adjusted^b^	
	OR	95% CI	OR	95% CI
Mean change in shoulder pain (for a 1-cm increase in VAS)	0.6	0.3 to 1.0	0.6	0.2 to 1.4
High FABQ-PA^c^	0.9	0.3 to 2.4	1.2	0.4 to 4.3
Interaction between change in shoulder pain and high FABQ-PA			0.3	0.0 to 1.9

*Abbreviations*: *CI* Confidence interval, *FABQ-PA* Fear-Avoidance Beliefs Questionnaire—Physical Activity, *OR* Odds ratio, *VAS* Visual Analogue Scale (ranging from 0 [no pain] to 10 [worst imaginable pain])

^a^High adherence was defined as ≥ 2 weekly exercise sessions

^b^Adjusted for age, sex, body mass index, smoking status, dominant-sided pain, baseline pain at rest, intervention group and an interaction term between change in shoulder pain and high FABQ-PA

^c^High FABQ-PA was defined as a baseline score > 14

## Discussion

This study demonstrated no associations between increased shoulder pain across an exercise session and lower subsequent exercise dose, regardless of the level of fear-avoidance beliefs. In addition, increased shoulder pain across exercise sessions together with high fear-avoidance beliefs did not influence adherence to the exercise programme.

Strengths of the study include the prospective design, the high participation (72%), the high data completeness and the repeated data collection. The main limitation was that the participants were informed that pain aggravation during exercise could be expected, and if the pain did not decrease within 1 h after exercise, they were advised to reduce the exercise dose in the subsequent exercise session. We did not specify the maximum tolerable pain aggravation, and it is reassuring that the exercise programme generally did not markedly aggravate shoulder pain (cf. the introduction). On the other hand, this information could have led to the low increase in pain across exercise sessions that we observed, i.e., low exposure contrast, and could have especially affected participants with a high FABQ-PA because they may be more likely to avoid exercise due to pain [13, 14]. Regarding FABQ-PA we did not observe such a pattern. High fear-avoidance beliefs might cause participants to drop out of an exercise intervention due to pain, but the mean FABQ-PA for participants included and those with missing data were comparable both in case of high and in case of low FABQ-PA. Therefore, we do not think that fear-avoidance beliefs increased the risk of attrition bias in our study. None of the main results were statistically significant, which might suggest that the study was underpowered. However, the mean differences were minimal for increase in shoulder pain, FABQ-PA and their interactions, so these factors did not seem to play any considerable role for subsequent exercise dose. Exercise dose and adherence were assessed by self-report, but we find it unlikely that under- or over-reporting of exercise efforts would be systematically related to increase in pain across an exercise session. Therefore, we do not think that the self-reported information biased our results.

Our study showed no indications of exercise progression despite all participants having a detailed description of the progression method in the exercise pamphlet. We are not aware of studies that have described progression in home-based settings, but in a study of 12 weeks’ supervised shoulder exercises, it was found that the resistance level increased by 74% [23].

Baseline FABQ-PA was comparable to previous studies of participants with shoulder complaints [32, 34]. In a study of participants with more than 6 months of neck or shoulder pain, persons with high fear-avoidance beliefs were more likely to drop out of the home-based exercise intervention [16]. In contrast, we found no association between FABQ-PA and adherence to the exercise programme. Participants in the just-mentioned study [16] had comparable baseline fear-avoidance beliefs (21 (0 to 16 scale)) and pain intensities (4 on the NRS) to our participants, but their median pain duration was 102 months (IQR 60 to 168 months), i.e. considerably longer than reported by the participants in the present study. Therefore, we think that the inclusion of patients with chronic pain in the previous study might explain why fear-avoidance beliefs played a part in that study but not in ours.

Our exercise programme was designed to support adherence. Adherence in our study was comparable with a previous study of home-based shoulder exercise [39], but higher adherence has also been reported; i.e., 88% completion of two daily sessions [40] and 96% completion of at least one daily session [41], compared with 39% completion of at least two weekly sessions in our study. A reason for our rather low adherence may be that in our participants, baseline pain was too low to motivate frequent exercise performance [12]. Our participants had not sought treatment for their complaints, whereas previous participants were in contact with the health care system [40, 41]. Another explanation could be lack of time because about 40% of the participants in one of the previous studies were on sick leave [40] compared with none of our participants. The other previous study did not inform about sick leave, but baseline pain was high (7 to 8 on the NRS) [41], indicating a probability of sick leave. Lack of time can decrease exercise motivation [12], and in line with this sick leave may enhance the motivation.

In the present study, participants with missing data seemed comparable to those included in the analyses, although they tended to be younger, more often men and more often smokers. Those with missing data seemed to have longer pain durations, but the difference only represented 10% of the total range. Due to the missing data, we were not able to tell whether these participants forgot to report their completed exercise sessions or whether they did not exercise, but we tend to assume the latter. This suggests that participants with the just-mentioned characteristics may need extra attention and guidance in relation to exercise therapy.

Employees could be included in the present study [19], if they had at least slight shoulder complaints (OSS ≤ 40), and baseline pain intensities at rest and during activity as well as changes across exercise sessions were generally low. We cannot rule out that higher increases in pain across an exercise session, depending on the severity of the shoulder disorder or the exercise programme, may lead to a subsequently reduced exercise dose. Thus, our results do not reveal whether an association exits between increases in higher pain intensities across an exercise session and subsequent exercise dose. Future studies of patients, e.g., in hospital departments of orthopaedic surgery, may examine whether this is the case.

## Conclusion

Increased shoulder pain across an exercise session was not a barrier for subsequent exercise dose nor exercise adherence, regardless of fear-avoidance beliefs among persons with slight shoulder complaints.

## Supplementary Information

**Additional file 1.**

## Data Availability

The datasets generated and analysed during the current study are not publicly accessible, but are available from the corresponding author on reasonable request and under standard conditions.

## Abbreviations

BMI: Body mass index
CI: Confidence interval
FABQ-PA: Fear-Avoidance Beliefs Questionnaire—Physical Activity
IQR: Interquartile range
MD: Mean difference
NRS: Numerical rating scale
OR: Odds ratio
OSS: Oxford Shoulder Score
SD: Standard deviation
VAS: Visual analogue scale
