# Supplementary material for: Increased shoulder pain across an exercise session and subsequent shoulder exercise: a prospective cohort study
Source: BMC Musculoskelet Disord. 2022 Jul 29;23:726. doi: 10.1186/s12891-022-05674-2 (PMC9336042; doi:10.1186/s12891-022-05674-2)
Supplement: Supplementary file 1 — Additional file 1. [file 12891_2022_5674_MOESM1_ESM.docx]

# Additional file

**Additional file 1:** An extract from a page in the exercise dairy


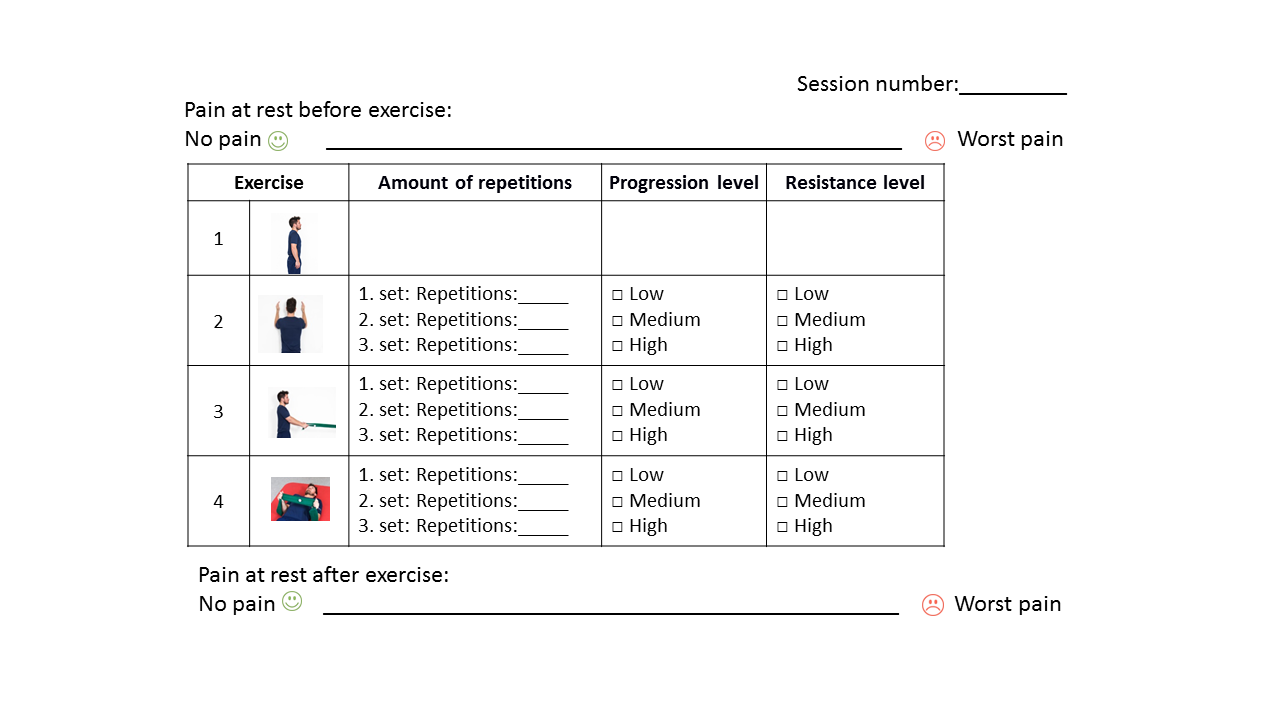


In the original exercise diaries, which were provided in Danish, the line for reporting pain before and after exercise was 10 cm from "No pain" to "Worst pain".
